# Impact of agro-forestry systems on the aroma generation of coffee beans

**DOI:** 10.3389/fnut.2022.968783

**Published:** 2022-08-04

**Authors:** Su Xu, Yuze Liu, Fengwei Ma, Ni Yang, Elias de Melo Virginio Filho, Ian Denis Fisk

**Affiliations:** ^1^Food and Pharmaceutical Engineering Institute, Guiyang University, Guiyang, China; ^2^Division of Food Sciences, University of Nottingham, Nottingham, United Kingdom; ^3^Centro Agronómico Tropical de Investigación y Enseñanza, CATIE, Turrialba, Costa Rica

**Keywords:** agro-forestry systems, shade trees, organic management, coffee color, aroma generation

## Abstract

A long experiment has been established since 2000 at CATIE (Tropical Agricultural Research and Higher Education Center), Turrialba, Costa Rica. Twenty agro-forestry systems with different shade types and managements (organic and non-organic) consisting of an incomplete randomized block-design with shade tree as main effect and subplots represented by management were set up. The effects of different managements and shade types on the aroma and color generation of roasted coffee beans were investigated. The total protein content was significantly higher (*P* < 0.05) under the intensive conventional (IC) (168 g/Kg) and intensive organic (IO) (167 g/Kg) managements than under the moderate conventional (MC) (153 g/Kg in IC vs. MC group, 157 g/Kg in MC vs. IO group). Comparing with the moderate conventional (MC) management, the intensive organic (IO) management had a stronger ability to generate more flavor and color. The total protein content was significantly higher (*P* < 0.05) under the full sun system (172 g/Kg) than under the shaded (159 g/Kg) and *Erythrina* system (155 g/Kg), under the service system (165 g/Kg) than under the timber system (146 g/Kg), under the legume timber system (170 g/Kg) than under the non-legume timber system (152 g/Kg). The full sun system had a greater flavor generation and color after roasting. Comparing with the timber system, the service system produced roasted beans with the more flavor and color. Comparing with the non-legume shade tree, the legume shade tree improved the performance of flavor and color in the roasted coffee beans.

## Introduction

Coffee is widely appreciated and consumed due to its pleasant flavor generated during the roasting process (1). In addition, expert coffee tasters assess the quality of coffee, to a greater extent, by its aroma and taste, and the highest quality coffee beans can obtain a considerable price premium (2). A large number of investigations, therefore, have been carried out to evaluate the types and quantities of volatile compounds as well as its formation principals in the roasted coffee beans. Most volatile compounds formed during roasting are derived from a range of chemical precursors in the green coffee beans, these include sugars, amino acids, organic acids and phenolic compounds, and these react through many complex reactions including the Maillard reaction, degradation of sugars and lipid oxidation (3–7). Nine specific pathways to generate the volatile compounds during roasting of coffee have been outlined by Dart and Nursten (2) in 1985. There were the Maillard reaction, Strecker degradation, trigonelline degradation, phenolic acids degradation, lipid degradation, sugar degradation, breakdown of sulphur amino acid, breakdown of hydroxyl amino acids and proline, and hydroxyproline degradation. Moreover, the dominating volatile compounds, according to their functional groups, have been divided into ten classes by Dart and Nursten (2). They included sulphur compounds, pyrazines, pyridines, pyrroles, oxazoles, furans, aldehydes, ketones, phenols, and other heterocyclic compounds.

There are many sourcess affecting the aroma generation of roasted coffee beans: the origin of coffee beans (i.e., Arabica, Robusta) may contribute to the differences (8); seasonal variation may also play an essential role in differences (9, 10); additionally, differences in geographical locations (11), differences in post-harvest processing (wet or dry processing) and aging before roasting also affect the final aroma generation (1). Furthermore, roasting profile (time-temperature) and the roaster models are aslo significant contributors to the differences of final coffee flavor (12, 13).

Coffee roasting plays an essential role in the formation of organoleptic properties including flavor and color. During the roasting process, moisture content decreases and many chemical reactions occur, along with crucial variations in terms of flavor, color, volume, weight, bean pop, pH, density and presence of volatile compounds (14).

Gas chromatograph is widely used to analyse the coffee volatile composition followed by mass spectrometer (15) or other specific detectors [flame ionization detectors (16), nitrogen-phosphorous detectors, photo-ionization detectors] due to its sensitivity and operability. It can separate and identify a complex mixture of aromas in one operation as well as quantify the volatiles at extremely low concentrations (2).

Numerous researches have been carried to identify the volatile compounds of coffee, explore the formation mechanisms of these pleasant aromas and investigate the relationship between the composition of green coffee beans and aroma generation of roasted coffee beans (17–21), but only a limited number of studies have actually connected these to the growth conditions, levels and types of fertilizers, the species of shade trees. As a result, it is necessary for coffee scientists to explore the interactions and detail the specific effects of fertilizer levels and shade types on aroma generation of roasted coffee beans. The aim of this research was to investigate the impact of agro-forestry systems including fertilizer levels and shade types on the aroma generation of roasted coffee bean.

## Materials and methods

### Agro-forestry methodologies

The experiment was established in 2000 at CATIE (Tropical Agricultural Research and Higher Education Center), Turrialba, Costa Rica (9°53'44” N, 83°40'7” W, CATIE, Turrialba, Costa Rica), which is defined as a low altitude (600 m above sea level), wet coffee zone without a marked dry season. Average annual rainfall, temperature, relative humidity and solar radiation was 2,915 mm/year, 22°C, 90.2%, and 15.9 MJ/m^2^/year (2000–2013).

Twenty agroforestry systems with different shade types and managements consisting of an incomplete randomized block-design with shade tree as main effect and subplots represented by management were set up (Table 1). For each system, three replicates were established. Shade type [initially 417 trees per ha-1 (6 × 4 m^2^ spacing)] consisted of timber and service tree species with contrasting characteristics (Table 2). Trees were progressively thinned to maintain a reasonable shade environment for coffee production (Table 3).

**Table 1:** Agroforestry systems with main plot (Shade type) and subplot (Management) treatments.

Shade types^*^	**E**	**T**	**C**	**C+T**	**E+T**	**C+E**	**Full Sun**
Managements^**^	IC	IC				IC	IC
	MC	MC	MC	MC	MC	MC	MC
	IO	IO	IO	IO	IO	IO	
	LO	LO				LO	

* *E, Erythrina poepiggiana; C, Chloroleucon eurycyclum; T, Terminalia amazonia*.

** *IC, Intensive conventional; MC, Moderate conventional; IO, Intensive organic, LO, Low organic; (n = 3)*.

**Table 2:** Characteristics of shade trees (22).

**Species**	**Phenology**	**Canopy**	**N-fixer**	**Use**
*Erythrina poepiggiana* (E)	Evergreen	Low compact	Yes	Service
*Chloroleucon eurycyclum* (C)	Deciduous^*^	High spreading	Yes	Timber
*Terminalia amazonia* (T)	Deciduous^*^	High compact	No	Timber

* * Deciduous for about 20–30 days per year*.

**Table 3:** Mean shade tree density after thinning.

**Agroforestry system**		**Tree density per ha** ^**−1**^		
**System**	**Tree species**	**2008**	**2011**	**2013**
**Monocultures**				
E	E	360	285	241
C	C	381	154	65
T	T	317	167	73
**Polycultures**				
C+E	C	183	100	45
	E	181	134	115
C+T	C	166	77	39
	T	170	77	34
E+T	E	147	143	109
	T	158	81	34

*E, Erythrina poepiggiana; C, Chloroleucon eurycyclum; T, Terminalia Amazonia*.

Intensive conventional (IC) *Erythrina* trees were biannually pollarded to a 1.8–2.0 m main trunk. Whilst this is normal practice in Costa Rica, Muschler (24) found that coffee quality benefited from increased *Erythrina* shade levels, therefore, for all the other treatments with *Erythrina*, trees were pollarded to 4 m leaving three branches for partial shade. Temporary shade was planted in the form of *Ricinus* in organic treatments. This took place 1 year after the coffee plants, to improve coffee plant survival and impede weed growth. Lower branches of the timber trees were pruned annually (year 1–7) to improve stem quality. In all pruning scenarios, pruning residuals from coffee trees and shade trees were left on the ground (trunks were removed). Management consisted of fertilization, weed, disease and pest control, detailed in Table 4.

**Table 4:** Mean input levels of fertilizers (Kg ha^−1^year^−1^) and weed/disease control since 2006, adapted from Haggar et al. (23) and Noponen et al. (25).

**Management**	Fertilization N:P:K ^**^	**Weed control**	**Disease/Pest control**
IC	287:20:150	Six^*^ herbicides	3–4^*^ Fungicides/ insecticides
MC	150:10:75	Five herbicides four manual	1–4 Fungicides/ insecticides as required
IO	248:205:326	Four Manual	Organic substances as required
LO	66:2:44	Four Manual	No

*IC, Intensive conventional, MC, Moderate conventional, IO, Intensive organic, LO, Low organic. IO fertilization: Chicken manure 10 t ha^−1^year^−1^and K-Mag (fertilizer)100 kg ha^−1^year^−1^; LO fertilization: Coffee pulp 5 t ha^−1^year^−1^*.

* *Number of treatments applied per year*.

** *Fertilization levels (Kg ha^−1^year^−1^) are 7 years means (2003–2009), from the second to fourth year LO systems received the same fertilization as IO ones, due to the site limitations that did not allow organic coffee to establish effectively with lower inputs*.

*Coffea Arabica* L. var. *Caturra*, was planted at 5000 holes ha^−1^ with dead plants replaced each year. Two plants per planting hole were planted (local practice) but were treated as one plant in every analysis. The distance between rows and holes were 2 m and 1 m. Sub-plots were 500–600 m^2^ of which the central 225–300 m^2^ was studied (100 coffee plants and 24 shade trees).

Coffee plants were manually pruned from 2004 leaving 1–4 resprouts per stump, according to the productive potential of each coffee resprout in the next harvest. Every coffee planting hole thus comprised 1–2 stumps and a total of 1–4 resprouts per stump.

### Coffee roasting

All green coffee beans harvested in different agro-forestry systems from CATIE, Costa Rica in both 2013 and 2014 were sent to Edgehill Coffee Company (Warwick, UK) for roasting. The coffee roaster (Roastilino, Fracino) with a small fluidized bed and a nominal capacity of about 200 g of green coffee beans was used and 50 g of green coffee beans were prepared and roasted. The air outlet temperature and roasting time were 166°C and 1 min 50 s, respectively. All coffee beans were taken past first crack and roasted to medium roasting. After roasting, 30 g of different roasted coffee beans were ground into powders using a mini chopper (CH180 mini chopper, 300 W, Kenwood) and a 710 μm sieve was used to obtain even coffee powder samples. All coffee beans and powders were stored at −20°C.

### Measurement of color

The colorimeter (ColorQuest XE, HunterLab Inc.) was used to measure the color of the coffee bean (25). The colorimeter was calibrated using the white plate and packed coffee powder was added to a transparent preservative film and then placed on the detection port to obtain data. Results were calculated by the equipment using the Hunter Lab color scale. In this scale, L ranges from 0 (black) to 100 (white), a indicates degree of greenness (for negative a values) and degree of redness (for positive a results), b axis also ranges from negative to positive values indicating, respectively, degree of blueness to yellowness.

### Measurement of total protein content

A bicinchoninic acid (BCA) assay kit (23225/23227, Thermo Scientific) was used to measure the total protein content (26).

0.1 g of coffee powders (wet basis, 11% water content) was weighed and 2 mL of chloroform was used to extract lipid with three replicates. After lipid extraction, the solid phase was dried at 60°C for 30 min and placed in a 15 mL centrifuge tube. After that, 1 mL of 2% sodium dodecyl sulfate (SDS) solution was added into the tube to eliminate interference from lipids and the tube was heated at 60°C in a water bath for 30 min. The sample was then vortexed for 1 min and centrifuged at 13,000 g for 3 min. And then, samples were filtered and diluted 100 times using 2% SDS.

According to the instructions of BCA protein assay kit, 0.1 mL of each standard and sample replicate was added into test tubes. 2.0 mL of the working reagent containing 50 parts of BCA Reagent A (sodium carbonate, sodium bicarbonate, bicinchoninic acid and sodium tartrate in 0.1 mol/L sodium hydroxide) and 1 part of BCA Reagent B (4% cupric sulfate) was added to each tube and mixed well. All tubes were covered and incubated at 37°C for 30 min, after incubation, tubes were cooled to room temperature. The absorbance of the samples was measured at 562 nm by the ultraviolet spectrophotometer (Evolution 350, Thermo Scientific) and subtracted the average 562 nm absorbance measurement of the Blank standard replicates. A standard curve was prepared by plotting the average blank-corrected 562 nm measurements for each bovin serum albumin (BSA) standard vs. its concentration in μg/mL. Finally, the standard curve was used to determine the protein concentration of each sample.

### Measurement of sucrose

The sucrose standard was prepared at a concentration of 10, 20, 40, and 50 mg/L. 0.1 g of coffee powder was placed in a 50 mL centrifuge tube with 20 mL of boiling water and vortexed for 5 min. Samples then were centrifuged at 1,600 g for 10 min at room temperature. After centrifugation, the liquid phase was transferred into a new glass vial. The above processes were repeated three times for extracting sucrose completely. The mixture was cooled to room temperature and then filtered using a syringe filter (0.45 μm, hydrophilic nylon syringe filter, Millipore Corporation). The final extract was diluted with water (1:1) prior to Liquid chromatography-mass spectrometry (LC/MS) analysis [the method was modified from Ky et al. (27), Mccusker et al. (28), Perrone et al. (29)].

The LC equipment (1,100 Series, Agilent) consisted of a degasser (G1322A, Agilent), a pump (G1312A, Agilent), an auto-sampler (G1313A, Agilent). This LC system was interfaced with a Quattro Ultima mass spectrometer (Micromass, UK Ltd.) fitted with an electrospray ion source.

The Kromasil 5 ODS (C18) column (250 × 3.20 mm, 5 μm, Phenomenex) was used to separate sucrose and the column was maintained at room temperature. The mobile phase consisted of 0.3% aqueous formic acid (eluent A) and methanol (eluent B) which was delivered at a flow rate of 0.4 ml/min. Initially the solvent was 25% B. After the injection, this proportion was changed immediately to 60% B until the end of the run at 8 min. Between injections, the column was re-equilibrated with 25% B for 4 min.

The electrospray ionization source was operated in the negative mode (3.5 KV) from 0.0 to 4.0 min to generate the formic acid adduct of sucrose (M+HCOO)^−^ and in the positive mode (3.5 KV) from 4.0 to 8.0 min to generate caffeine (M+H)^+^ ions. Desolvation gas (N_2_) flow rate was 500 L/h and desolvation temperature was 400°C. The mass spectrometer was operated in the selected ion recording (SIR) model to measure sucrose. Identification of compounds was performed by comparing retention time and molecular weight of the respective standards. Quantification was achieved by comparing peak areas of samples and standards using Masslynx software (version 4.0, Waters) (the method was modified from 24 and 25).

### Measurement of volatile compounds

2 g of each coffee sample powders and 2.5 μL of 1% heptanone (internal standard) were placed GC vials for analysis (30).

A GC-ISQ (Thermo Electron Corporation Inc.) was used to detect the volatile compounds of coffee in full scan mode over the mass range 20–200 m/z (ion source 200°C). The sample was incubated at 55°C for 5 min with shaking using the autosampler. A 50/30 μm DVB/CAR/PDMS (Divinylbenzene/Carboxen/Polydimethylsiloxane) was the fiber sampled the volatiles for 3.5 min followed by a 1.5 min desorption (inlet temperature 200°C). A splitless mode was used and the constant carrier pressure was at 103 kPa.

The GC oven was held at 40°C for 5 min, and then increased from 40 to 240°C at a rate of 5°C/min, and held at 240°C for 2 min. Separation was carried out on a ZB-WAX Capillary GC Column (Length 30 m, inner diameter 0.25 mm, and film thickness 0.25 μm; Phenomenex Inc., Macclesfield, UK). The identity and quantity of volatile compounds was determined using Thermo Xcalibur software. The mass spectrum was used to identify the volatile compounds by comparing with the library (NIST/EPA/NIH Mass Spectral Library. Version 2.0, Faircom Corporation, U.S.) in Xcalibur software. The peak areas of compounds and the internal standard were determined using Thermo Xcalibur software (30). The following formula could be used to calculate the concentration of volatile compounds:


$$\begin{array}{l} The\;concentration\;of\;volatile\;compounds\;\left(mg/kg\right)\\ =\frac{Peak\;area\;of\;volatile\;compound\times Amount\;of\;internal\;standard\left(mg\right)}{Peak\;area\;of\;internal\;standard\times Dry\;weight\;of\;coffee\;powder\left(kg\right)\;} \end{array}$$

### Statistical analysis

The experimental design consisted of shade types as main plot and subplots represented by managements but with an unbalanced structure since not all managements were represented under all Shade types (Table 1). Therefore, the specific pre-planned contrast models (Modified from 19) (Table 5) were used to compare the differences between different managements and shade types.

**Table 5:** Principal contrasts used in the analysis of shade type and management effects.

**Contrast**	**Treatments compared**
**Management**	
IC vs. MC	IC(FS, E, T, CE) vs. MC(FS, E, T, CE)
MC vs. IO	MC(E, T, C, CE, CT, ET) vs. IO(E, T, C, CE, CT, ET)
IO vs. LO	IO(E, CE) vs. LO(E, CE)
IC vs. IO	IC(E, T, CE) vs. IO(E, T, CE)
**Shade type**	
FS vs. shaded Erythrina vs. FS^*^	FS(IC, MC) vs. E(IC, MC) + T(IC, MC) + CE(IC, MC) E(IC, MC) vs. FS(IC, MC)
Service vs. timber trees	E(MC, IO) vs. T(MC, IO) + C(MC, IO) + TC(MC, IO)
Legume timber vs. non-legume timber	C(MC, IO) vs. T(MC, IO)

*IC, Intensive conventional; MC, Moderate conventional; IO, Intensive organic; LO, Low organic; FS, Full sun. E, Erythrina poepiggiana; C, Chloroleucon eurycyclum; T, Terminalia Amazonia; CE, Chloroleucon eurycyclum and Erythrina poepiggiana; CT, Chloroleucon eurycyclum and Terminalia Amazonia; ET, Erythrina poepiggiana and Terminalia Amazonia*.

* *Erythrina was regarded as a low canopy tree with low shade cover and compared with full sun*.

Data was analyzed using One-Way ANOVA contrasts model with SPSS statistics software (Version 22nd, IBM), treatment as contrast factor to analyze eight contrast groups as shown in Table 5. Significant value (*P* < 0.05) in contrast tests was used to decide if there was a significant difference in each contrast model and the standard error of each contrast could be obtained from contrast tests table. In addition, the mean value of every management and shade type under each contrast model was calculated.

## Results and discussion

### Impact of fertilizer levels and shade types on the total protein and sucrose contents of green coffee bean

During the coffee roasting progress, carbohydrates especially sucrose are degraded, dehydrated and interacted with protein to generate a large number of volatile compounds (31, 32), which contributes to the formation of organoleptic properties of roasted coffee beans including flavor, aroma and color (14). As a result, the sucrose and protein were analyzed due to the main contribution to the generation of volatile compounds.

The results of total protein content were shown in Table 6. The total protein content was significantly lower (*P* < 0.05) under the MC (153, 11.3 g/Kg) than under the IC (168, 12.2 g/Kg). Furthermore, the total protein content was significantly higher (*P* < 0.05) under the IO (167, 12.4 g/Kg) than under the MC (157, 11.8 g/Kg). Nevertheless, no significant differences (*P* > 0.05) were detected in the total protein content between the IO and LO, and the IC and IO.

**Table 6:** Contrast results for total protein and sucrose contents (g/Kg) by dry weight of green coffee beans.

**Contrast**	**Total protein** **(g/Kg)**			**Sucrose** **(g/Kg)**		
**Managements**	**Mean**	**S.E._D_**	***p*-value**	**Mean**	**S.E._D_**	***p*-value**
IC vs. MC	167.62	17.25	**0.001**	91.52	9.12	0.544
	152.53			90.09		
MC vs. IO	157.11	21.31	**0.01**	88.12	11.25	0.053
	166.51			92.25		
IO vs. LO	162.39	12.18	0.94	86.97	6.91	0.915
	161.92			87.33		
IC vs. IO	166.92	14.73	0.414	89.89	8.21	0.328
	162.79			87.22		
**Shade types**						
FS vs. S	171.88	29.87	**0.011**	91.73	16.05	0.647
	159.05			90.49		
E vs. FS	155.36	12.11	**<0.001**	90.36	7.36	0.68
	171.78			91.73		
Ser. vs. Tim.	165.33	30.24	**<0.001**	88.50	16.18	0.984
	146.29			88.56		
LT vs. NLT	170.33	12.21	**<0.001**	84.21	6.87	0.077
	152.26			90.14		

*Values were presented as mean, standard error of the contrast difference (S.E.D), and significance of the difference (P-value), P-values <0.05 were shown in bold. IC, Intensive conventional; MC, Moderate conventional; IO, Intensive organic; LO, Low organic; FS, Full sun; S, Shaded; E, Erythrina poepiggiana; Ser., Service, Tim, Timber; LT, Legume timber; NLT, Non-legume timber*.

For the different shade types, the total protein content of green coffee beans was significantly higher (*P* < 0.05) under the full sun system (172 g/Kg) than under the shaded system (159 g/Kg) and the *Erythrina* system (155 g/Kg). In addition, the total protein content of green coffee beans was significantly higher (*P* < 0.05) under the service system (165 g/Kg) than under the timber system (146 g/Kg), while it was significantly lower (*P* < 0.05) under the non-legume timber system (152 g/Kg) than under the legume timber system (170 g/Kg).

The sucrose content was shown in the Table 6. There were no significant differences (*P* > 0.05) detected between the different managements and shade types.

The total protein content was significantly higher (*P* < 0.05) under the IC and IO managements than under the MC due to the higher input amount of fertilizers especially the nitrogen. After a long period management (about 13 years), the IO management had a similar total protein content in the green coffee beans to the IC management and even a significantly higher (*P* < 0.05) value than the MC management. Nonetheless, there was no significant difference (*P* > 0.05) in the total protein content between the IO and LO system. When compared with the IO management, the LO management was combined with the legume species *Erythrina* and *Chloroleucon*. On the one hand, the legume species can replenish the lack of N_2_ due to the low input level by N_2_ fixation (33); on the other hand, the high pruning intensity like *Erythrina* may compensate the lower external inputs, as the leaves and branches pruned from the *Erythrina* shade tree were left on the field (22, 34).

The result of the effect of different shade types on the total protein content suggested that light intensity was the main factor to drive the protein generation in the green coffee beans in our experiment, as the protein biosynthesis in the ribosome is adenosine triphosphate (ATP) dependent (35). As a result, the total protein content was significantly higher (*P* < 0.05) under the full sun system than under the shaded and *Erythrina* system due to the higher shade cover and lower light intensity in the shaded and *Erythrina* systems, moreover, it was also significantly higher (*P* < 0.05) under the service system than under the timber system due to the lower shade cover and higher light intensity in the service system (the *Erythrina* was the only service tree in our experiment). Additionally, the total protein content in the green coffee beans was significantly higher (*P* < 0.05) under the legume timber system than under the non-legume timber system due to the N_2_ fixation of the legume species to supply the organic N_2_ for the coffee plant to synthesize the amino acids and protein.

It is well-known that the inorganic carbon is converted to the organic carbon in the plant mainly through photosynthesis, and also, the plant can also absorb the organic carbon fixed by fungi as well as from organic matters on the field (36, 37). In addition, photosynthesis is affected by light intensity, carbon dioxide concentration, water availability, temperature (38, 39). For each contrast group of different managements in our experiment, the shade types have been fixed. Thus, it could be assumed that light intensity and temperature were the same when comparing the different managements in each contrast group. Different managements had no effect on the carbon dioxide concentration due to the same atmospheric conditions. Furthermore, Costa Rica site was considered as the wet coffee zone, which had the adequate water resource. Therefore, no significant differences (*P* > 0.05) were detected in the sucrose content between the different managements.

In our experiment, there were no significant differences (*P* > 0.05) found in the sucrose content between the full sun and shaded system, service and timber, and the legume timber and non-legume timber system, which demonstrated that the light intensity, shaded cover and the N_2_ fixation of shade tree were not the main factors to affect the sucrose content of green coffee beans.

### Impact of different fertilizer levels and shade types on the aroma and the color generation of roasted coffee beans

**Figure 1:**
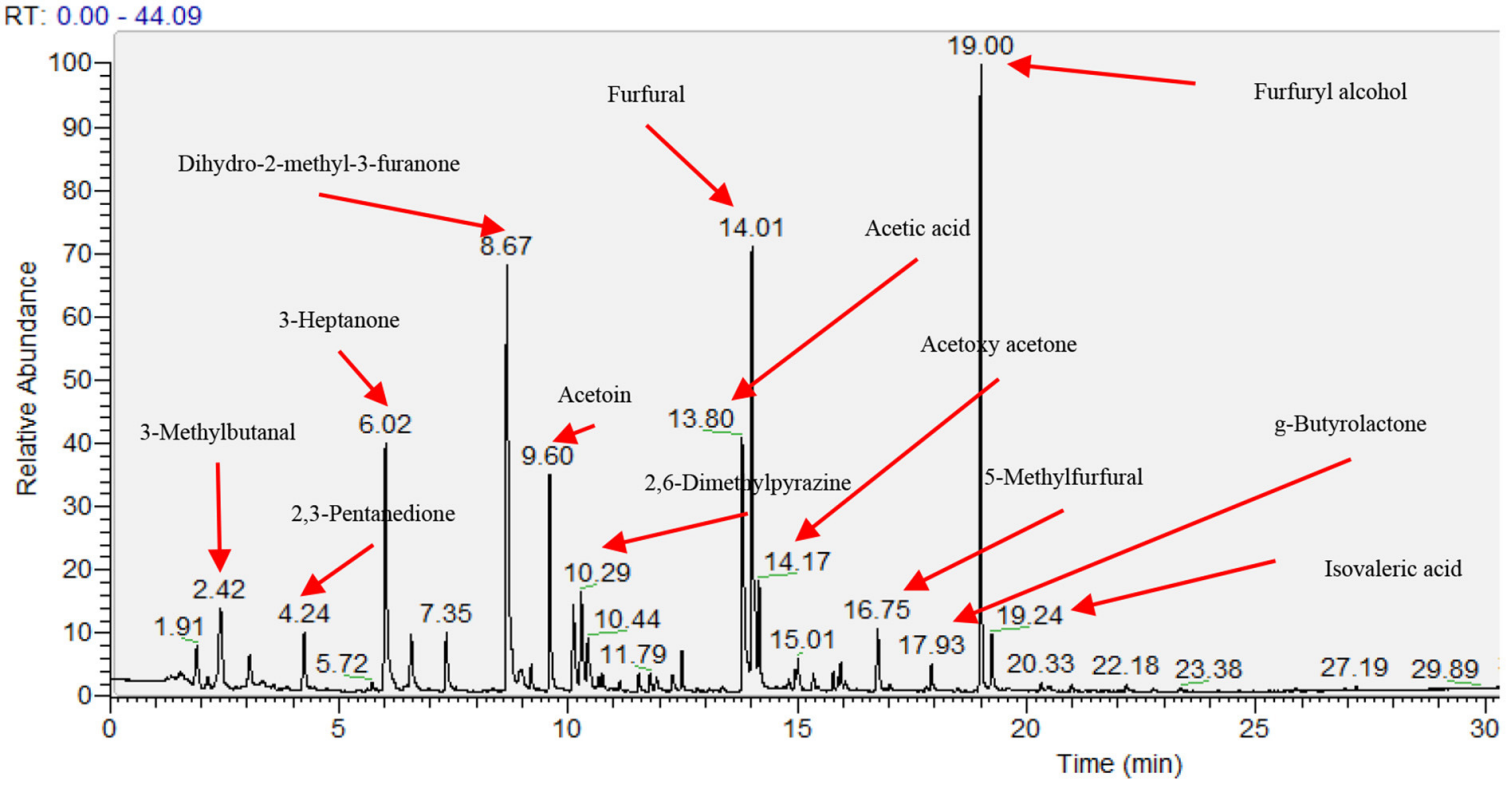
Gas chromatogram of head space of roasted coffee powders on ZB-WAX capillary column. The results showed the coffee beans from the intensive conventional management combined with the shade tree *Erythrina poepiggiana*.

Sixty-four volatile aroma compounds, which had previously been identified in coffee, were detected in the roasted coffee beans (30, 40). These aroma compounds were classified into thirteen groups based on their chemical attributes (three alcohols, four aldehydes, 11 ketones, three acids, one ether, two esters, 14 pyrazines, two pyridines, seven pyrroles, two sulfides, eight furans, five phenolic compounds, and 2 N-containing heterocyclic compounds), and their odors and functional group were described (Table 7). Figure 1 showed the gas chromatogram of roasted coffee beans and identified some main peaks of volatile aroma compounds based on the library (NIST/EPA/NIH Mass Spectral Library. Version 2.0, Faircom Corporation, U.S.). The quantitative data sets of the volatile aroma compounds measured in the current work can be found in Supporting Information.

**Table 7:** Detection of sixty-four volatile aroma compounds in the roasted coffee beans in the different agricultural treatments.

	**Aroma compound**	**ID**	**KI**	**Literature KI**	**Odor description**	**Functional group**
1	2,5-Dimethylfuran	m, r	721	706	Ethereal	Furan
2	2-Methylfuran	m, r	893	888	Chocolate	Furan
3	2-Methylbutanal	m, r	918	930	Malty	Aldehyde
4	3-Methylbutanal	m, r	950	934	Malty	Aldehyde
5	2,3-Butanedione	m, r	983	1,000	Buttery, cheesy	Ketone
6	Dimethyl-Disulphide	m, r	1,053	1,050	Onion	Sulphide
7	Hexanal	m, r	1,063	1,064	Grassy, green oily	Aldehyde
8	2,3-Pentanedione	m, r	1,078	1,072	Oily buttery	Ketone
9	2-Vinylfuran	m, r	1,112	1,096	Ethereal, rum, cocoa note	Furan
10	2,3-Hexanedione	m, r	1,154	1,138	Buttery, cheesy, sweet, creamy	Ketone
11	1-Methylpyrrole	m, r	1,171	1,168	White bread, woody	Pyrrole
12	2,4,5-Trimethyloxazole	m, r	1,219	1,200	Green, woody, musty	Heterocyclic N
13	Pyridine	m, r	1,225	1,213	Bitter, astringent, roasted, burnt	Pyridine
14	2-Pentylfuran	m, r	1,239	1,235	Fruity, green	Fruan
15	Furfuryl methyl ether	m, r	1,262	1,243	Nutty, rich, phenolic	Ether
16	Dihydro-2-methyl-3-furanone	m, r	1,267	1,246	Sweet, roasted	Ketone
17	2-Methylpyrazine	m, r	1,282	1,297	Nutty, roasted, chocolate	Pyrazine
18	4-Methylthiazole	m, r	1,335	1,312	Nutty, green	Heterocyclic N
19	Acetoin	m, r	1,348	-	Buttery, creamy	Ketone
20	2,5-Dimethylpyrazine	m, r	1,370	1,357	Nutty, roasted, grassy, corn	Pyrazine
21	2,6-Dimethylpyrazine	m, r	1,375	1,362	Nutty, sweet, fried	Pyrazine
22	2-Ethyl-3-methylpyrazine	m, r	1,382	1,363	Nutty	Pyrazine
23	2-Methyl-2-cyclopentenone	m, r	1,388	1,366	Fruity	Ketone
24	2-Ethylpyrazine	m, r	1,391	1,370	Nutty, roasted	Pyrazine
25	2,3-Dimethylpyrazine	m,r	1,395	1,383	Nutty, roasted, green	Pyrazine
26	2,3,5-Trimethylpyrazine	m, r	1,410	1,395	Nutty, roasted	Pyrazine
27	2-Ethyl-5-methylpyrazine	m, r	1,416	1,397	Nutty, roasted	Pyrazine
28	2-Ethyl-6-methylpyrazine	m, r	1,426	1,420	Roasted, hazelnut-like	Pyrazine
29	Propyl pyrazine	m, r	1,437	1,428	Green	Pyrazine
30	Vinyl pyrazine	m, r	1,446	1,434	Nutty, green	Pyrazine
31	2,6-Diethylpyrazine	m, r	1,458	1,444	Nutty, roasted	Pyrazine
32	Acetic acid	m, r	1,471	1,454	Sour	Acid
33	Furfural	m, r	1,479	1,462	Bread, almond, sweet	Aldehyde
34	2-Ethyl-3,5-dimethylpyrazine	m, r	1,483	1,464	Nutty	Pyrazine
35	Acetoxy acetone	m, r	1,491	1,469	Buttery	Ketone
36	2-Ethyl-3,6-dimethylpyrazine	m, r	1,498	1,480	Nutty, roasted	Pyrazine
37	2-Fufurylmethylsulfide	m, r	1,506	-	Alliaceous, sulfurous	Sulphide
38	2-Acetylfuran	m, r	1,514	1,500	Balsamic-sweet	Furan
39	Pyrrole	m, r	1,518	1,512	Nutty, hay-like, herbaceous	Pyrrole
40	2,3-Dimethyl-2-cyclopentenone	m, r	1,523	1,524	Grassy, bitter	Ketone
41	Propionic acid	m, r	1,536	1,527	Sour	Acid
42	Acetoxy-2-butanone	m, r	1,546	-	Sour	Ketone
43	2-Furfurylacetate	m, r	1,552	1,566	Fruity, green	Ester
44	5-Methylfurfural	m, r	1,562	1,566	Sweet, caramel, bready	Furan
45	3-Methylpyrrole	m, r	1,574	1,569	Woody	Pyrrole
46	2-Acetylpyridine	m, r	1,588	1,602	Popcorn type, corn type	Pyridine
47	1-Methyl-2-formylpyrrole	m, r	1,593	1,626	Bread, burnt, caramel	Pyrrole
48	2-Furfuryl-5-methylfuran	m, r	1,647	1,659	Chocolate	Furan
49	g-Butyrolactone	m, r	1,658	-	Creamy, milky	Ester
50	Furfuryl alcohol	m, r	1,671	1,662	Burnt	Alcohol
51	Isovaleric acid	m, r	1,681	1,682	Cheesy	Acid
52	2,5-Dihydrofuranone	m, r	1,749	1,767	Caramel	Ketone
53	1-Furfurylpyrrole	m, r	1,814	1,822	Vegetable	Pyrrole
54	Guaiacol	m, r	1,843	1,855	Phenolic, woody	Phenolic
55	2-Thiophenemethanol	m,r	1,872	1,890	savory	Alcohol
56	Phenylethyl alcohol	m, r	1,902	1,896	Floral	Alcohol
57	2-Acetylpyrrole	m, r	1,953	1,949	White bread	Pyrrole
58	Difurfuryl ether	m,r	1,991	1,977	Coffee-like, toasted odour	Ether
59	Phenol	m, r	2,011	-	Smoky	Phenolic
60	4-Ethylguaiacol	m,r	2,042	2,034	Phenolic	Phenolic
61	2-Formylpyrrole	m, r	2,048	2,036	White bread, jasmine rice	Pyrrole
62	Furaneol	m, r	2,053	2,037	Fruity	Ketone
63	p-Cresol	m, r	2,091	2,078	Phenolic-type	Phenolic
64	2-Methoxy-4-vinylguaiacol	m, r	2,112	-	Woody, smoky	Phenolic

*ID, Identification method; m, mass spectrum based on Mass library - NIST/EPA/NIH Mass Spectral Library. Version 2.0, Faircom Corporation, U.S.; r, identified by retention index; KI, Kovats index calculated by n-alkanes on the ZB-WAX Capillary GC Column (Length 30 m, inner diameter 0.25 mm, and film thickness 0.25 μm; Phenomenex Inc., Macclesfield, UK); Literature KI was collected from NIST database: webbook.nist.gov; Odor Description: data was collected from odor database: www.flavornet.org; origin-scifinder.cas.org)*.

**Figure 2:**
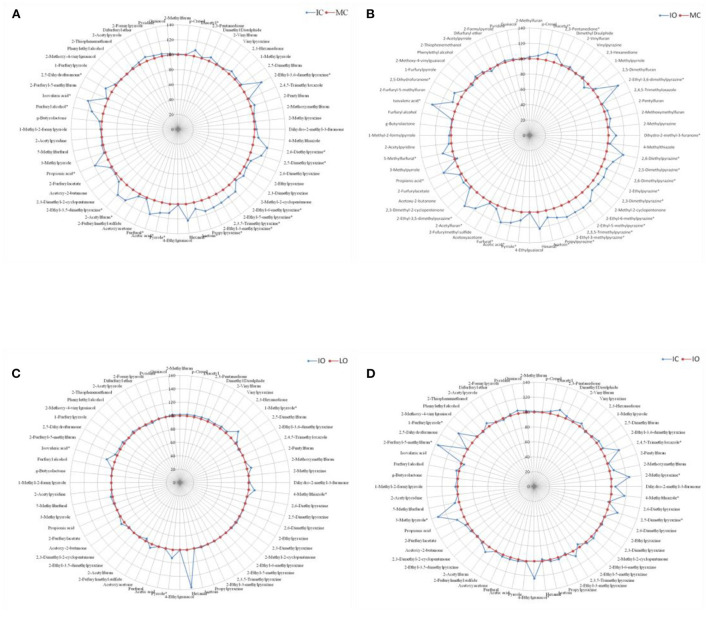
Comparison of volatile aroma compounds generation in roasted coffee beans between the different managements, results were presented as the mean value of three replicates. **(A)** Intensive conventional (IC) vs. moderate conventional (MC). **(B)** Intensive organic (IO) vs. moderate conventional (MC). **(C)** Intensive organic (IO) vs. low organic (LO). **(D)** Intensive conventional (IC) vs. intensive organic (IO). *represented significant differences (*P* < 0.05) of volatile compounds between different groups.

**Figure 3:**
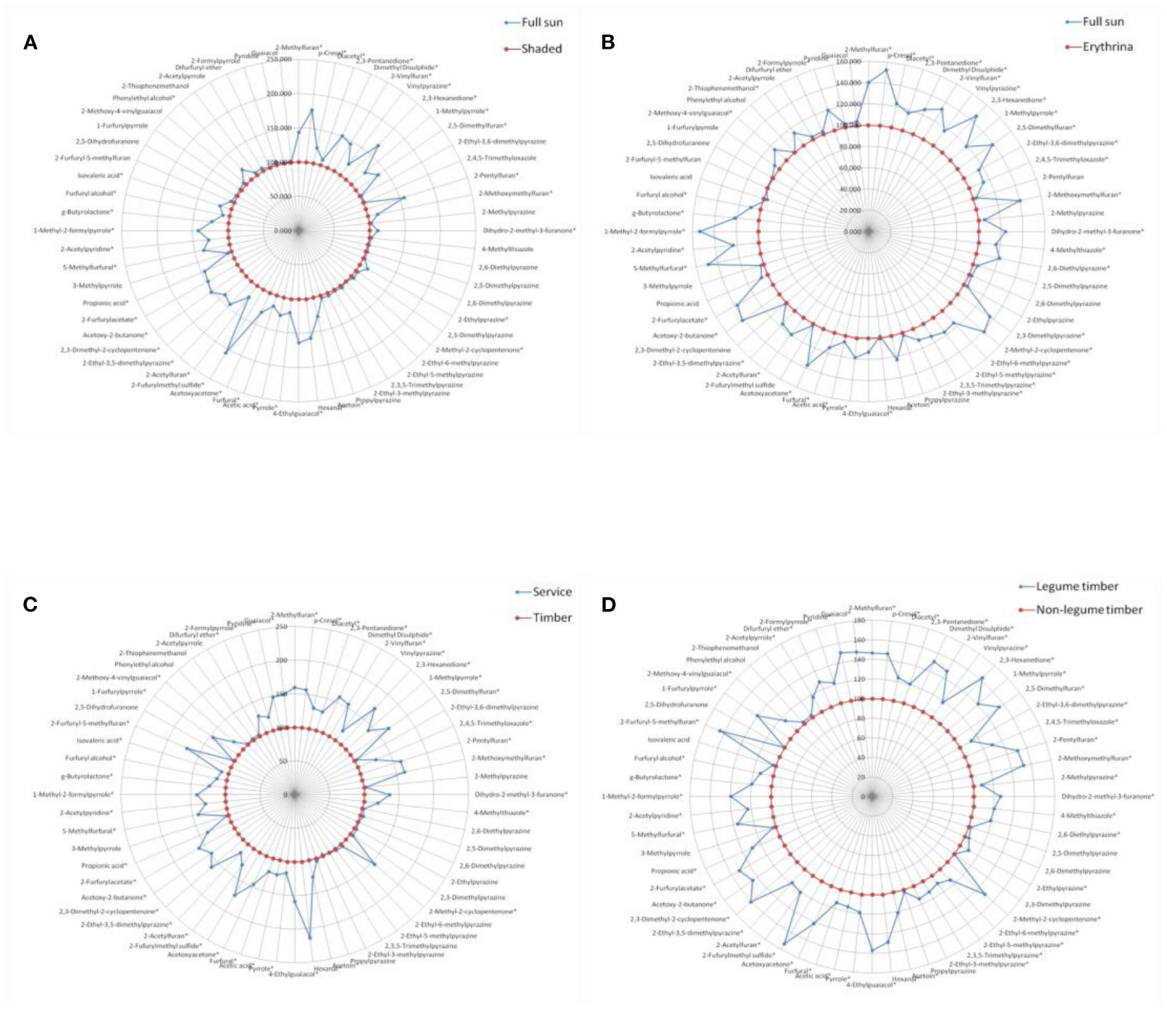
Comparison of volatile aroma compounds generation in roasted coffee beans between the different shade types, results were presented as the mean value of three replicates. **(A)** Full sun vs. shaded. **(B)** Full sun vs. *Erythrina*. **(C)** Service vs. timber. **(D)** Legume timber vs. non-legume timber. *represented significant differences (*P* < 0.05) of volatile compounds between different groups.

For each contrast model, the fertilizer level with the relatively lower amount of volatile compounds generation was assumed as the control group and the other one with the relatively higher amount of volatile compounds generation was supposed to be the non-control group. The values of these volatile compounds in the control group were defined as 100. The ratio of the non-control group to the control group were calculated, and then, the final values of these volatile compounds in the non-control group were expressed as the ratio multiplied by 100. For example, in the Figure 2A, the MC with the relatively lower volatile compounds generation was assumed as the control group and showed as the circle with round markers at 100%, while the IC with the relatively higher abundance of volatile compounds was defined as the non-control group and showed as the spider diagram with diamond markers. Although sixty-four volatile compounds were detected in our experiment, only sixty volatile compounds were displayed in Figures 2, 3, as there were sno significant differences (*P* > 0.05) in the amounts of these four volatile compounds (2-Methylbutanal, Isovaleraldehyde, Phenol and Furaneol) between the different fertilizer levels and shade types.

Figure 2 showed the effect of different managements on the aroma generation of roasted coffee beans. The relative abundance of twenty volatile compounds were significantly higher (*P* < 0.05) in the roasted coffee beans under the IC management than in those roasted coffee beans under the MC management (Figure 2A, more details see Table 1 in supplementary). The amounts of twenty-five volatile compounds were significantly higher (*P* < 0.05) in the roasted coffee beans under the IO management than in those roasted coffee beans under the MC management (Figure 2B, more details see Table 2 in supplementary file). Nevertheless, only five volatile compounds had significantly higher (*P* < 0.05) amounts in the roasted coffee beans under the IO management than in those roasted coffee beans under the LO management (Figure 2C, more details see Table 3 in supplementary file). Moreover, there were eight volatile compounds with a significantly higher (*P* < 0.05) amount in the roasted coffee beans under the IC management than in those roasted coffee beans under the IO management (Figure 2D, more details see Table 4 in supplementary file).

Similarly, for each contrast model, the shade type with the relatively lower volatile compounds generation was assumed as the control group and the other one with the relatively higher volatile compounds generation was supposed to be the non-control group. The values of these volatile compounds in the control group were defined as 100. The ratio of the non-control group to the control group was calculated, and then, the final values of these volatile compounds in the non-control group were expressed as the ratio multiplied by 100.

Figure 3 showed the effect of different shade types on the aroma generation of roasted coffee beans. Thirty-six volatile compounds had a significantly higher (*P* < 0.05) amounts in the roasted coffee beans under the full sun system than in those roasted coffee beans under the shaded system (Figure 3A, more details see Table 5 in supplementary file). The amounts of forty volatile compounds were significantly higher (*P* < 0.05) in the roasted coffee beans under the full sun system than in those roasted coffee beans under the Erythrina system (Figure 3B, more details see Table 6 in supplementary file). Additionally, there were forty-three volatile compounds with a significantly higher (*P* < 0.05) amount in the roasted coffee beans under the service shade tree system than in those roasted coffee beans under the timber shade tree system (Figure 3C, more details see Table 7 in supplementary file). The amounts of fifty-one volatile compounds were significantly higher (*P* < 0.05) in the roasted coffee beans under the legume timber shade tree system than in those roasted coffee beans under the non-legume timber shade tree system (Figure 3D, more details see Table 8 in supplementary file).

As mentioned in the methods, L value ranges from 0 to 100, which represents the relevant color is from black (0) to white (100). As a result, the color turns darker with the decreasing of L value. Table 8 showed the effects of fertilizer levels and shade tree types on the color of roasted coffee beans.

**Table 8:** Contrast results for the color of roasted coffee beans.

**Contrast**	***L*** **-value**		
	**Mean**	**S.E._D_**	***p*-value**
**Managements**			
IC vs. MC	50.77	2.01	**0.042**
	53.96		
MC vs. IO	54.02	2.21	**0.006**
	50.26		
IO vs. LO	53.04	1.33	0.085
	54.52		
IC vs. IO	53.16	2.21	0.102
	54.03		
**Shade types**			
FS vs. S	52.97	3.05	**0.016**
	56.82		
E vs. FS	57.70	1.21	**<0.001**
	52.97		
Ser.v. Tim.	50.53	3.11	**<0.001**
	56.98		
LT vs. NLT	52.09	1.27	**<0.001**
	59.48		

*Values were presented as mean, standard error of the contrast difference (S.E._D_) and significance of the difference (P-value), P-values <0.05 were shown in bold. IC, Intensive conventional; MC, Moderate conventional; IO, Intensive organic; LO, Low Organic; FS, Full sun; S, Shaded; E, Erythrina poepiggiana; Ser., Service; Tim, Timber; LT, Legume timber; NLT, Non-legume timber*.

With regards to the different fertilizer levels, *L*-values of roasted coffee beans were significantly lower (*P* < 0.05) under the IC (51) and the IO (50) management than under the MC (54) management. As a result, the color of roasted coffee beans was significantly darker (*P* < 0.05) under the IC and the IO management than under the MC management. Nevertheless, no significant differences (*P* > 0.05) were detected in the L values between the IO and LO, and the IC and IO.

In terms of shade types, the roasted coffee beans under the full sun system (53) had a significantly lower (*P* < 0.05) L-value than under the shaded (57) and *Erythrina* (58) systems. Thus, the color of roasted coffee beans was significantly darker (*P* < 0.05) under the full sun system than under the shaded and *Erythrina* systems. Furthermore, the L value of roasted coffee beans was significantly lower (*P* < 0.05) under the service shade tree system (51) than under the timber shade tree system (57), while there was a significantly higher (*P* < 0.05) L value detected in the roasted coffee beans under the non-legume timber shade tree system (59) than under the legume timber shade tree system (52). Therefore, the color of roasted coffee beans was significantly darker (*P* < 0.05) under the service shade tree system than under the timber shade tree system, whilst the roasted coffee beans under the non-legume timber shade tree system had a significantly lighter (*P* < 0.05) color than under the legume shade tree system.

Although many complex chemical reactions such as the degradation of sugar and acids occur during coffee bean roasting progress, Maillard reaction is obviously one of the most important chemical reactions to promote the formation of coffee flavor and color (2, 41). Maillard reaction is related to the interactions between reducing sugars and amino acids (42). Even though there is a large number of polysaccharides present in the green coffee beans (43), most carbohydrates are relatively stable to roasting (44, 45) and therefore it is the relative abundance of free or available sugars drives the thermal flavor generation. Moreover, on roasting, sucrose is rapidly degraded and its content dramatically decreases by about 97% (46). As a result, during coffee bean roasting, the reducing sugars and amino acids, as the main precursors of Maillard reaction, are mainly derived from the decomposition of sucrose and protein in the green coffee beans, respectively. Furthermore, the melanoidins, which contributes to the color formation of roasted coffee beans, are derived from Maillard reaction and dehydration of sucrose (47). Therefore, it is proposed that the contents of sucrose and total protein in the green coffee beans are responsible for the formation of coffee flavor and color.

There were no significant differences (*P* > 0.05) detected in the sucrose content of green coffee beans among the different fertilizer levels and shade types (see Table 6). Nonetheless, significant differences (*P* < 0.05) were found in the total protein content of green coffee beans among the different fertilizer levels and shade types (see Table 6).

In terms of the different fertilizer levels, the total protein content of green coffee beans was significantly lower (*P* < 0.05) under the MC management than under the IC and IO management. This explains why the roasted coffee beans under MC management presented a significantly lower (*P* < 0.05) number of volatile compounds and lighter color than those roasted coffee beans under the IC and IO management.

With regards to the different shade types, the amounts of volatile compounds in the roasted coffee beans were significantly higher (*P* < 0.05) and the color of roasted coffee beans was significantly darker (*P* < 0.05) under the full sun system than under the shaded and *Erythrina* system due to a significantly higher (*P* < 0.05) total protein content of green coffee beans in the full sun system. Similarly, due to the significantly higher (*P* < 0.05) total protein content of green coffee beans under the service and legume timber shade tree system, the roasted coffee beans under the service and legume timber shade tree system had a significantly higher (*P* < 0.05) number of volatile compounds and significantly darker (*P* < 0.05) color than those roasted coffee beans under the timber and non-legume timber shade tree system, respectively.

## Conclusion

To sum up, in our experiment, the total protein content of green coffee beans was significantly affected by the fertilizer levels and shade types, while there was no significant effect on the sucrose content of green coffee beans. As a result, the total protein content was the main factor to drive the formation of aroma and color of roasted coffee beans. As has been discussed, the green coffee beans under the intensive conventional (IC) and intensive organic (IO) management had a significantly higher (*P* < 0.05) total protein content than those under the moderate conventional (MC) management due to the higher fertilizer input amount, especially the nitrogen. Thus, the higher N input amount could increase the protein generation in the green coffee beans and further improve the formation of final flavor and color of roasted coffee beans. Furthermore, the legume shade tree could improve the protein generation in the green coffee beans by fixing more N_2_ and further promote the formation of final flavor and color of roasted coffee beans. Compared with shaded system, the light intensity of full sun system was significant higher, which improved the protein biosynthesis in the ribosome, and finally, promoted the formation of flavor and color of roasted coffee beans.

## Data availability statement

The original contributions presented in the study are included in the article/Supplementary material, further inquiries can be directed to the corresponding author.

## Author contributions

SX, IF, and EV conceived the experiments. SX, YL, and FM drafted the manuscript. SX conducted all the experiments. NY helped to discuss the results and perfected the language assisted with the structure elucidation and manuscript revision. IF and EV designed and supervised the research and revised the manuscript. All authors have read and agreed to the published version of the manuscript.

## Funding

This research was financially supported by Science and Technology Fund Project of Guizhou (Qian Ke He Zhicheng [2022] Zhongdian No. 015), Youth Science and Technology Talent Development Project of Education Department of Guizhou Province (Qian Jiao He KY Zi [2018]303), the Special Funding of Guiyang Science, and Technology Bureau and Guiyang University (GYU-KY-[2021]).

## Conflict of interest

The authors declare that the research was conducted in the absence of any commercial or financial relationships that could be construed as a potential conflict of interest.

## Publisher's note

All claims expressed in this article are solely those of the authors and do not necessarily represent those of their affiliated organizations, or those of the publisher, the editors and the reviewers. Any product that may be evaluated in this article, or claim that may be made by its manufacturer, is not guaranteed or endorsed by the publisher.
