# Supplementary material for: Impact of agro-forestry systems on the aroma generation of coffee beans
Source: Front Nutr. 2022 Aug 4;9:968783. doi: 10.3389/fnut.2022.968783 (PMC9386424; doi:10.3389/fnut.2022.968783)
Supplement: Supplementary file 1 [file Table_1.docx]

**Table 1 The quantitative data for volatile aroma compounds in the contrast group IC vs MC**

|  | IC  (mg/kg) | MC  (mg/kg) |
| --- | --- | --- |
| 2-Methylfuran | 0.0185 | 0.0184 |
| p-Cresol | 0.0069 | 0.0069 |
| Diacetyl | 0.0529 | 0.0488 |
| 2,3-Pentanedione | 0.0927 | 0.0918 |
| Dimethyl Disulphide | 0.0027 | 0.0026 |
| 2-Vinylfuran | 0.0054 | 0.0053 |
| Vinylpyrazine | 0.0021 | 0.0021 |
| 2,3-Hexanedione | 0.0049 | 0.0047 |
| 1-Methylpyrrole | 0.0095 | 0.0093 |
| 2,5-Dimethylfuran | 0.0024 | 0.0023 |
| 2-Ethyl-3,6-dimethylpyrazine | 0.0030 | 0.0024 |
| 2,4,5-Trimethyloxazole | 0.0008 | 0.0008 |
| 2-Pentylfuran | 0.0003 | 0.0002 |
| 2-Methoxymethylfuran | 0.0015 | 0.0015 |
| 2-Methylpyrazine | 0.3159 | 0.3121 |
| Dihydro-2-methyl-3-furanone | 0.0520 | 0.0505 |
| 4-Methylthiazole | 0.0025 | 0.0023 |
| 2,6-Diethylpyrazine | 0.0005 | 0.0004 |
| 2,5-Dimethylpyrazine | 0.0361 | 0.0316 |
| 2,6-Dimethylpyrazine | 0.0710 | 0.0684 |
| 2-Ethylpyrazine | 0.0383 | 0.0372 |
| 2,3-Dimethylpyrazine | 0.0128 | 0.0121 |
| 2-Methyl-2-cyclopentenone | 0.0015 | 0.0014 |
| 2-Ethyl-6-methylpyrazine | 0.0136 | 0.0121 |
| 2-Ethyl-5-methylpyrazine | 0.0089 | 0.0080 |
| 2,3,5-Trimethylpyrazine | 0.0101 | 0.0090 |
| 2-Ethyl-3-methylpyrazine | 0.0080 | 0.0071 |
| Propylpyrazine | 0.0298 | 0.0260 |
| Acetoin | 0.0259 | 0.0238 |
| Hexanal | 0.0007 | 0.0005 |
| 4-Ethylguaiacol | 0.0001 | 0.0001 |
| Pyrrole | 0.0090 | 0.0080 |
| Acetic acid | 0.3961 | 0.3471 |
| Furfural | 0.4162 | 0.3492 |
| Acetoxyacetone | 0.1122 | 0.1102 |
| 2-Fufurylmethyl sulfide | 0.0009 | 0.0008 |
| 2-Acetylfuran | 0.0347 | 0.0296 |
| 2-Ethyl-3,5-dimethylpyrazine | 0.0007 | 0.0006 |
| 2,3-Dimethyl-2-cyclopentenone | 0.0005 | 0.0004 |
| Acetoxy-2-butanone | 0.0160 | 0.0158 |
| 2-Furfurylacetate | 0.0235 | 0.0231 |
| Propionic acid | 0.0105 | 0.0089 |
| 3-Methylpyrrole | 0.0002 | 0.0002 |
| 5-Methylfurfural | 0.0792 | 0.0777 |
| 2-Acetylpyridine | 0.0005 | 0.0005 |
| 1-Methyl-2-formylpyrrole | 0.0031 | 0.0030 |
| g-Butyrolactone | 0.0093 | 0.0090 |
| Furfuryl alcohol | 0.2179 | 0.1891 |
| Isovaleric acid | 0.0287 | 0.0232 |
| 2-Furfuryl-5-methylfuran | 0.0001 | 0.0001 |
| 2,5-Dihydrofuranone | 0.0057 | 0.0052 |
| 1-Furfurylpyrrole | 0.0011 | 0.0011 |
| 2-Methoxy-4-vinylguaiacol | 0.0009 | 0.0009 |
| Phenylethyl alcohol | 0.0002 | 0.0002 |
| 2-Thiophenemethanol | 0.0003 | 0.0003 |
| 2-Acetylpyrrole | 0.0018 | 0.0017 |
| Difurfuryl ether | 0.0001 | 0.0001 |
| 2-Formylpyrrole | 0.0021 | 0.0021 |
| Pyridine | 0.0675 | 0.0653 |
| Guaiacol | 0.0004 | 0.0004 |
