# Supplementary material for: Impact of agro-forestry systems on the aroma generation of coffee beans
Source: Front Nutr. 2022 Aug 4;9:968783. doi: 10.3389/fnut.2022.968783 (PMC9386424; doi:10.3389/fnut.2022.968783)
Supplement: Supplementary file 2 [file Table_2.docx]

**Table 2 The quantitative data for volatile aroma compounds in the contrast group MC vs IO**

|  | MC  (mg/kg) | IO  (mg/kg) |
| --- | --- | --- |
| 2-Methylfuran | 0.0192 | 0.0197 |
| p-Cresol | 0.0079 | 0.0083 |
| Diacetyl | 0.0506 | 0.0559 |
| 2,3-Pentanedione | 0.0871 | 0.0960 |
| Dimethyl Disulphide | 0.0030 | 0.0030 |
| 2-Vinylfuran | 0.0058 | 0.0059 |
| Vinylpyrazine | 0.0023 | 0.0022 |
| 2,3-Hexanedione | 0.0050 | 0.0051 |
| 1-Methylpyrrole | 0.0099 | 0.0092 |
| 2,5-Dimethylfuran | 0.0025 | 0.0026 |
| 2-Ethyl-3,6-dimethylpyrazine | 0.0025 | 0.0032 |
| 2,4,5-Trimethyloxazole | 0.0009 | 0.0009 |
| 2-Pentylfuran | 0.0003 | 0.0003 |
| 2-Methoxymethylfuran | 0.0016 | 0.0017 |
| 2-Methylpyrazine | 0.3174 | 0.3222 |
| Dihydro-2-methyl-3-furanone | 0.0491 | 0.0540 |
| 4-Methylthiazole | 0.0024 | 0.0025 |
| 2,6-Diethylpyrazine | 0.0004 | 0.0005 |
| 2,5-Dimethylpyrazine | 0.0318 | 0.0355 |
| 2,6-Dimethylpyrazine | 0.0669 | 0.0715 |
| 2-Ethylpyrazine | 0.0367 | 0.0399 |
| 2,3-Dimethylpyrazine | 0.0122 | 0.0132 |
| 2-Methyl-2-cyclopentenone | 0.0015 | 0.0016 |
| 2-Ethyl-6-methylpyrazine | 0.0127 | 0.0146 |
| 2-Ethyl-5-methylpyrazine | 0.0082 | 0.0095 |
| 2,3,5-Trimethylpyrazine | 0.0092 | 0.0106 |
| 2-Ethyl-3-methylpyrazine | 0.0073 | 0.0085 |
| Propylpyrazine | 0.0265 | 0.0299 |
| Acetoin | 0.0251 | 0.0274 |
| Hexanal | 0.0005 | 0.0006 |
| 4-Ethylguaiacol | 0.0001 | 0.0001 |
| Pyrrole | 0.0082 | 0.0091 |
| Acetic acid | 0.3654 | 0.4246 |
| Furfural | 0.3743 | 0.4458 |
| Acetoxyacetone | 0.1185 | 0.1239 |
| 2-Fufurylmethyl sulfide | 0.0009 | 0.0009 |
| 2-Acetylfuran | 0.0323 | 0.0380 |
| 2-Ethyl-3,5-dimethylpyrazine | 0.0006 | 0.0007 |
| 2,3-Dimethyl-2-cyclopentenone | 0.0005 | 0.0005 |
| Acetoxy-2-butanone | 0.0172 | 0.0179 |
| 2-Furfurylacetate | 0.0250 | 0.0256 |
| Propionic acid | 0.0098 | 0.0118 |
| 3-Methylpyrrole | 0.0002 | 0.0002 |
| 5-Methylfurfural | 0.0785 | 0.0885 |
| 2-Acetylpyridine | 0.0006 | 0.0006 |
| 1-Methyl-2-formylpyrrole | 0.0033 | 0.0034 |
| g-Butyrolactone | 0.0098 | 0.0098 |
| Furfuryl alcohol | 0.2126 | 0.2158 |
| Isovaleric acid | 0.0208 | 0.0271 |
| 2-Furfuryl-5-methylfuran | 0.0001 | 0.0001 |
| 2,5-Dihydrofuranone | 0.0054 | 0.0060 |
| 1-Furfurylpyrrole | 0.0012 | 0.0012 |
| 2-Methoxy-4-vinylguaiacol | 0.0010 | 0.0010 |
| Phenylethyl alcohol | 0.0002 | 0.0002 |
| 2-Thiophenemethanol | 0.0003 | 0.0003 |
| 2-Acetylpyrrole | 0.0020 | 0.0019 |
| Difurfuryl ether | 0.0001 | 0.0001 |
| 2-Formylpyrrole | 0.0023 | 0.0023 |
| Pyridine | 0.0690 | 0.0701 |
| Guaiacol | 0.0005 | 0.0005 |
