# Supplementary material for: Impact of agro-forestry systems on the aroma generation of coffee beans
Source: Front Nutr. 2022 Aug 4;9:968783. doi: 10.3389/fnut.2022.968783 (PMC9386424; doi:10.3389/fnut.2022.968783)
Supplement: Supplementary file 3 [file Table_3.docx]

**Table 3 The quantitative data for volatile aroma compounds in the contrast group IO vs LO**

|  | IO  (mg/kg) | LO  (mg/kg) |
| --- | --- | --- |
| 2-Methylfuran | 0.0200 | 0.0197 |
| p-Cresol | 0.0075 | 0.0073 |
| Diacetyl | 0.0517 | 0.0505 |
| 2,3-Pentanedione | 0.0913 | 0.0890 |
| Dimethyl Disulphide | 0.0029 | 0.0028 |
| 2-Vinylfuran | 0.0056 | 0.0055 |
| Vinylpyrazine | 0.0022 | 0.0021 |
| 2,3-Hexanedione | 0.0050 | 0.0049 |
| 1-Methylpyrrole | 0.0103 | 0.0090 |
| 2,5-Dimethylfuran | 0.0025 | 0.0025 |
| 2-Ethyl-3,6-dimethylpyrazine | 0.0031 | 0.0031 |
| 2,4,5-Trimethyloxazole | 0.0009 | 0.0009 |
| 2-Pentylfuran | 0.0003 | 0.0003 |
| 2-Methoxymethylfuran | 0.0015 | 0.0014 |
| 2-Methylpyrazine | 0.3230 | 0.3231 |
| Dihydro-2-methyl-3-furanone | 0.0504 | 0.0498 |
| 4-Methylthiazole | 0.0026 | 0.0024 |
| 2,6-Diethylpyrazine | 0.0005 | 0.0005 |
| 2,5-Dimethylpyrazine | 0.0345 | 0.0347 |
| 2,6-Dimethylpyrazine | 0.0715 | 0.0710 |
| 2-Ethylpyrazine | 0.0392 | 0.0388 |
| 2,3-Dimethylpyrazine | 0.0131 | 0.0128 |
| 2-Methyl-2-cyclopentenone | 0.0014 | 0.0014 |
| 2-Ethyl-6-methylpyrazine | 0.0140 | 0.0140 |
| 2-Ethyl-5-methylpyrazine | 0.0091 | 0.0091 |
| 2,3,5-Trimethylpyrazine | 0.0104 | 0.0103 |
| 2-Ethyl-3-methylpyrazine | 0.0083 | 0.0083 |
| Propylpyrazine | 0.0264 | 0.0260 |
| Acetoin | 0.0250 | 0.0249 |
| Hexanal | 0.0007 | 0.0005 |
| 4-Ethylguaiacol | 0.0001 | 0.0001 |
| Pyrrole | 0.0094 | 0.0083 |
| Acetic acid | 0.3593 | 0.3555 |
| Furfural | 0.3610 | 0.3604 |
| Acetoxyacetone | 0.1094 | 0.1028 |
| 2-Fufurylmethyl sulfide | 0.0008 | 0.0009 |
| 2-Acetylfuran | 0.0309 | 0.0310 |
| 2-Ethyl-3,5-dimethylpyrazine | 0.0007 | 0.0007 |
| 2,3-Dimethyl-2-cyclopentenone | 0.0005 | 0.0005 |
| Acetoxy-2-butanone | 0.0160 | 0.0152 |
| 2-Furfurylacetate | 0.0230 | 0.0227 |
| Propionic acid | 0.0094 | 0.0094 |
| 3-Methylpyrrole | 0.0002 | 0.0002 |
| 5-Methylfurfural | 0.0746 | 0.0726 |
| 2-Acetylpyridine | 0.0005 | 0.0005 |
| 1-Methyl-2-formylpyrrole | 0.0032 | 0.0032 |
| g-Butyrolactone | 0.0088 | 0.0087 |
| Furfuryl alcohol | 0.1933 | 0.1913 |
| Isovaleric acid | 0.0229 | 0.0204 |
| 2-Furfuryl-5-methylfuran | 0.0001 | 0.0001 |
| 2,5-Dihydrofuranone | 0.0053 | 0.0052 |
| 1-Furfurylpyrrole | 0.0011 | 0.0011 |
| 2-Methoxy-4-vinylguaiacol | 0.0010 | 0.0010 |
| Phenylethyl alcohol | 0.0002 | 0.0002 |
| 2-Thiophenemethanol | 0.0003 | 0.0003 |
| 2-Acetylpyrrole | 0.0018 | 0.0018 |
| Difurfuryl ether | 0.0001 | 0.0001 |
| 2-Formylpyrrole | 0.0021 | 0.0021 |
| Pyridine | 0.0738 | 0.0735 |
| Guaiacol | 0.0004 | 0.0004 |
