# Supplementary material for: Impact of agro-forestry systems on the aroma generation of coffee beans
Source: Front Nutr. 2022 Aug 4;9:968783. doi: 10.3389/fnut.2022.968783 (PMC9386424; doi:10.3389/fnut.2022.968783)
Supplement: Supplementary file 4 [file Table_4.docx]

**Table 4 The quantitative data for volatile aroma compounds in the contrast group IC vs IO**

|  | IC  (mg/kg) | IO  (mg/kg) |
| --- | --- | --- |
| 2-Methylfuran | 0.0195 | 0.0193 |
| p-Cresol | 0.0078 | 0.0078 |
| Diacetyl | 0.0509 | 0.0492 |
| 2,3-Pentanedione | 0.0901 | 0.0833 |
| Dimethyl Disulphide | 0.0029 | 0.0028 |
| 2-Vinylfuran | 0.0058 | 0.0058 |
| Vinylpyrazine | 0.0023 | 0.0022 |
| 2,3-Hexanedione | 0.0051 | 0.0052 |
| 1-Methylpyrrole | 0.0098 | 0.0097 |
| 2,5-Dimethylfuran | 0.0025 | 0.0024 |
| 2-Ethyl-3,6-dimethylpyrazine | 0.0032 | 0.0031 |
| 2,4,5-Trimethyloxazole | 0.0009 | 0.0007 |
| 2-Pentylfuran | 0.0003 | 0.0003 |
| 2-Methoxymethylfuran | 0.0016 | 0.0015 |
| 2-Methylpyrazine | 0.3307 | 0.2668 |
| Dihydro-2-methyl-3-furanone | 0.0518 | 0.0491 |
| 4-Methylthiazole | 0.0026 | 0.0022 |
| 2,6-Diethylpyrazine | 0.0005 | 0.0005 |
| 2,5-Dimethylpyrazine | 0.0363 | 0.0319 |
| 2,6-Dimethylpyrazine | 0.0639 | 0.0643 |
| 2-Ethylpyrazine | 0.0412 | 0.0405 |
| 2,3-Dimethylpyrazine | 0.0137 | 0.0137 |
| 2-Methyl-2-cyclopentenone | 0.0015 | 0.0014 |
| 2-Ethyl-6-methylpyrazine | 0.0148 | 0.0145 |
| 2-Ethyl-5-methylpyrazine | 0.0099 | 0.0104 |
| 2,3,5-Trimethylpyrazine | 0.0109 | 0.0101 |
| 2-Ethyl-3-methylpyrazine | 0.0092 | 0.0094 |
| Propylpyrazine | 0.0255 | 0.0249 |
| Acetoin | 0.0242 | 0.0237 |
| Hexanal | 0.0005 | 0.0005 |
| 4-Ethylguaiacol | 0.0001 | 0.0001 |
| Pyrrole | 0.0087 | 0.0086 |
| Acetic acid | 0.3668 | 0.3584 |
| Furfural | 0.3765 | 0.3663 |
| Acetoxyacetone | 0.1130 | 0.1099 |
| 2-Fufurylmethyl sulfide | 0.0009 | 0.0009 |
| 2-Acetylfuran | 0.0315 | 0.0291 |
| 2-Ethyl-3,5-dimethylpyrazine | 0.0007 | 0.0007 |
| 2,3-Dimethyl-2-cyclopentenone | 0.0005 | 0.0005 |
| Acetoxy-2-butanone | 0.0168 | 0.0165 |
| 2-Furfurylacetate | 0.0246 | 0.0239 |
| Propionic acid | 0.0099 | 0.0090 |
| 3-Methylpyrrole | 0.0002 | 0.0002 |
| 5-Methylfurfural | 0.0798 | 0.0757 |
| 2-Acetylpyridine | 0.0006 | 0.0006 |
| 1-Methyl-2-formylpyrrole | 0.0034 | 0.0033 |
| g-Butyrolactone | 0.0091 | 0.0090 |
| Furfuryl alcohol | 0.2006 | 0.1939 |
| Isovaleric acid | 0.0215 | 0.0224 |
| 2-Furfuryl-5-methylfuran | 0.0001 | 0.0001 |
| 2,5-Dihydrofuranone | 0.0054 | 0.0053 |
| 1-Furfurylpyrrole | 0.0012 | 0.0010 |
| 2-Methoxy-4-vinylguaiacol | 0.0010 | 0.0010 |
| Phenylethyl alcohol | 0.0001 | 0.0001 |
| 2-Thiophenemethanol | 0.0003 | 0.0003 |
| 2-Acetylpyrrole | 0.0020 | 0.0020 |
| Difurfuryl ether | 0.0001 | 0.0001 |
| 2-Formylpyrrole | 0.0022 | 0.0022 |
| Pyridine | 0.0730 | 0.0700 |
| Guaiacol | 0.0005 | 0.0005 |
