# Supplementary material for: Impact of agro-forestry systems on the aroma generation of coffee beans
Source: Front Nutr. 2022 Aug 4;9:968783. doi: 10.3389/fnut.2022.968783 (PMC9386424; doi:10.3389/fnut.2022.968783)
Supplement: Supplementary file 5 [file Table_5.docx]

**Table 5 The quantitative data for volatile aroma compounds in the contrast group full sun vs shaded**

|  | Full Sun  (mg/kg) | Shaded  (mg/kg) |
| --- | --- | --- |
| 2-Methylfuran | 0.0211 | 0.0147 |
| p-Cresol | 0.0083 | 0.0047 |
| Diacetyl | 0.0538 | 0.0437 |
| 2,3-Pentanedione | 0.0977 | 0.0903 |
| Dimethyl Disulphide | 0.0031 | 0.0020 |
| 2-Vinylfuran | 0.0061 | 0.0042 |
| Vinylpyrazine | 0.0022 | 0.0018 |
| 2,3-Hexanedione | 0.0053 | 0.0032 |
| 1-Methylpyrrole | 0.0096 | 0.0076 |
| 2,5-Dimethylfuran | 0.0027 | 0.0020 |
| 2-Ethyl-3,6-dimethylpyrazine | 0.0028 | 0.0028 |
| 2,4,5-Trimethyloxazole | 0.0009 | 0.0009 |
| 2-Pentylfuran | 0.0003 | 0.0002 |
| 2-Methoxymethylfuran | 0.0016 | 0.0014 |
| 2-Methylpyrazine | 0.3221 | 0.3152 |
| Dihydro-2-methyl-3-furanone | 0.0552 | 0.0498 |
| 4-Methylthiazole | 0.0025 | 0.0025 |
| 2,6-Diethylpyrazine | 0.0005 | 0.0004 |
| 2,5-Dimethylpyrazine | 0.0339 | 0.0334 |
| 2,6-Dimethylpyrazine | 0.0686 | 0.0678 |
| 2-Ethylpyrazine | 0.0389 | 0.0348 |
| 2,3-Dimethylpyrazine | 0.0132 | 0.0128 |
| 2-Methyl-2-cyclopentenone | 0.0016 | 0.0015 |
| 2-Ethyl-6-methylpyrazine | 0.0136 | 0.0137 |
| 2-Ethyl-5-methylpyrazine | 0.0088 | 0.0085 |
| 2,3,5-Trimethylpyrazine | 0.0100 | 0.0099 |
| 2-Ethyl-3-methylpyrazine | 0.0079 | 0.0076 |
| Propylpyrazine | 0.0274 | 0.0266 |
| Acetoin | 0.0266 | 0.0208 |
| Hexanal | 0.0008 | 0.0005 |
| 4-Ethylguaiacol | 0.0001 | 0.0000 |
| Pyrrole | 0.0087 | 0.0073 |
| Acetic acid | 0.3991 | 0.3157 |
| Furfural | 0.4109 | 0.3564 |
| Acetoxyacetone | 0.1214 | 0.0934 |
| 2-Fufurylmethyl sulfide | 0.0009 | 0.0004 |
| 2-Acetylfuran | 0.0349 | 0.0292 |
| 2-Ethyl-3,5-dimethylpyrazine | 0.0006 | 0.0004 |
| 2,3-Dimethyl-2-cyclopentenone | 0.0005 | 0.0003 |
| Acetoxy-2-butanone | 0.0176 | 0.0115 |
| 2-Furfurylacetate | 0.0243 | 0.0164 |
| Propionic acid | 0.0110 | 0.0076 |
| 3-Methylpyrrole | 0.0002 | 0.0002 |
| 5-Methylfurfural | 0.0811 | 0.0592 |
| 2-Acetylpyridine | 0.0006 | 0.0005 |
| 1-Methyl-2-formylpyrrole | 0.0032 | 0.0023 |
| g-Butyrolactone | 0.0093 | 0.0075 |
| Furfuryl alcohol | 0.2090 | 0.1914 |
| Isovaleric acid | 0.0266 | 0.0228 |
| 2-Furfuryl-5-methylfuran | 0.0001 | 0.0001 |
| 2,5-Dihydrofuranone | 0.0057 | 0.0056 |
| 1-Furfurylpyrrole | 0.0012 | 0.0011 |
| 2-Methoxy-4-vinylguaiacol | 0.0010 | 0.0010 |
| Phenylethyl alcohol | 0.0002 | 0.0001 |
| 2-Thiophenemethanol | 0.0003 | 0.0003 |
| 2-Acetylpyrrole | 0.0019 | 0.0018 |
| Difurfuryl ether | 0.0001 | 0.0001 |
| 2-Formylpyrrole | 0.0022 | 0.0021 |
| Pyridine | 0.0706 | 0.0698 |
| Guaiacol | 0.0005 | 0.0005 |
