# Supplementary material for: Impact of agro-forestry systems on the aroma generation of coffee beans
Source: Front Nutr. 2022 Aug 4;9:968783. doi: 10.3389/fnut.2022.968783 (PMC9386424; doi:10.3389/fnut.2022.968783)
Supplement: Supplementary file 6 [file Table_6.docx]

**Table 6 The quantitative data for volatile aroma compounds in the contrast group *Erythrina* vs full sun**

|  | *Erythrina*  (mg/kg) | Full sun  (mg/kg) |
| --- | --- | --- |
| 2-Methylfuran | 0.0166 | 0.0233 |
| p-Cresol | 0.0055 | 0.0084 |
| Diacetyl | 0.0429 | 0.0526 |
| 2,3-Pentanedione | 0.0753 | 0.0882 |
| Dimethyl Disulphide | 0.0025 | 0.0031 |
| 2-Vinylfuran | 0.0043 | 0.0058 |
| Vinylpyrazine | 0.0019 | 0.0022 |
| 2,3-Hexanedione | 0.0033 | 0.0048 |
| 1-Methylpyrrole | 0.0081 | 0.0097 |
| 2,5-Dimethylfuran | 0.0021 | 0.0030 |
| 2-Ethyl-3,6-dimethylpyrazine | 0.0026 | 0.0030 |
| 2,4,5-Trimethyloxazole | 0.0007 | 0.0008 |
| 2-Pentylfuran | 0.0002 | 0.0002 |
| 2-Methoxymethylfuran | 0.0014 | 0.0019 |
| 2-Methylpyrazine | 0.2913 | 0.3081 |
| Dihydro-2-methyl-3-furanone | 0.0458 | 0.0568 |
| 4-Methylthiazole | 0.0021 | 0.0024 |
| 2,6-Diethylpyrazine | 0.0004 | 0.0005 |
| 2,5-Dimethylpyrazine | 0.0313 | 0.0325 |
| 2,6-Dimethylpyrazine | 0.0654 | 0.0670 |
| 2-Ethylpyrazine | 0.0358 | 0.0371 |
| 2,3-Dimethylpyrazine | 0.0090 | 0.0123 |
| 2-Methyl-2-cyclopentenone | 0.0013 | 0.0018 |
| 2-Ethyl-6-methylpyrazine | 0.0118 | 0.0136 |
| 2-Ethyl-5-methylpyrazine | 0.0075 | 0.0088 |
| 2,3,5-Trimethylpyrazine | 0.0087 | 0.0099 |
| 2-Ethyl-3-methylpyrazine | 0.0070 | 0.0078 |
| Propylpyrazine | 0.0269 | 0.0276 |
| Acetoin | 0.0212 | 0.0261 |
| Hexanal | 0.0005 | 0.0005 |
| 4-Ethylguaiacol | 0.0000 | 0.0001 |
| Pyrrole | 0.0077 | 0.0092 |
| Acetic acid | 0.3698 | 0.3915 |
| Furfural | 0.3511 | 0.4027 |
| Acetoxyacetone | 0.0999 | 0.1368 |
| 2-Fufurylmethyl sulfide | 0.0007 | 0.0008 |
| 2-Acetylfuran | 0.0304 | 0.0363 |
| 2-Ethyl-3,5-dimethylpyrazine | 0.0006 | 0.0007 |
| 2,3-Dimethyl-2-cyclopentenone | 0.0004 | 0.0004 |
| Acetoxy-2-butanone | 0.0134 | 0.0190 |
| 2-Furfurylacetate | 0.0164 | 0.0226 |
| Propionic acid | 0.0097 | 0.0103 |
| 3-Methylpyrrole | 0.0002 | 0.0002 |
| 5-Methylfurfural | 0.0541 | 0.0805 |
| 2-Acetylpyridine | 0.0005 | 0.0006 |
| 1-Methyl-2-formylpyrrole | 0.0021 | 0.0033 |
| g-Butyrolactone | 0.0075 | 0.0091 |
| Furfuryl alcohol | 0.1925 | 0.2104 |
| Isovaleric acid | 0.0241 | 0.0233 |
| 2-Furfuryl-5-methylfuran | 0.0001 | 0.0001 |
| 2,5-Dihydrofuranone | 0.0056 | 0.0057 |
| 1-Furfurylpyrrole | 0.0011 | 0.0011 |
| 2-Methoxy-4-vinylguaiacol | 0.0009 | 0.0011 |
| Phenylethyl alcohol | 0.0002 | 0.0002 |
| 2-Thiophenemethanol | 0.0003 | 0.0003 |
| 2-Acetylpyrrole | 0.0019 | 0.0019 |
| Difurfuryl ether | 0.0001 | 0.0001 |
| 2-Formylpyrrole | 0.0019 | 0.0023 |
| Pyridine | 0.0653 | 0.0673 |
| Guaiacol | 0.0004 | 0.0005 |
