# Supplementary material for: Impact of agro-forestry systems on the aroma generation of coffee beans
Source: Front Nutr. 2022 Aug 4;9:968783. doi: 10.3389/fnut.2022.968783 (PMC9386424; doi:10.3389/fnut.2022.968783)
Supplement: Supplementary file 7 [file Table_7.docx]

**Table 7 The quantitative data for volatile aroma compounds in the contrast group service shade trees vs timber shade trees**

|  | Service  (mg/kg) | Timber  (mg/kg) |
| --- | --- | --- |
| 2-Methylfuran | 0.0218 | 0.0137 |
| p-Cresol | 0.0084 | 0.0054 |
| Diacetyl | 0.0567 | 0.0432 |
| 2,3-Pentanedione | 0.1003 | 0.0777 |
| Dimethyl Disulphide | 0.0031 | 0.0019 |
| 2-Vinylfuran | 0.0061 | 0.0039 |
| Vinylpyrazine | 0.0022 | 0.0019 |
| 2,3-Hexanedione | 0.0055 | 0.0032 |
| 1-Methylpyrrole | 0.0099 | 0.0070 |
| 2,5-Dimethylfuran | 0.0028 | 0.0017 |
| 2-Ethyl-3,6-dimethylpyrazine | 0.0026 | 0.0025 |
| 2,4,5-Trimethyloxazole | 0.0008 | 0.0007 |
| 2-Pentylfuran | 0.0003 | 0.0002 |
| 2-Methoxymethylfuran | 0.0017 | 0.0010 |
| 2-Methylpyrazine | 0.3163 | 0.3068 |
| Dihydro-2-methyl-3-furanone | 0.0566 | 0.0415 |
| 4-Methylthiazole | 0.0025 | 0.0021 |
| 2,6-Diethylpyrazine | 0.0004 | 0.0004 |
| 2,5-Dimethylpyrazine | 0.0329 | 0.0315 |
| 2,6-Dimethylpyrazine | 0.0678 | 0.0673 |
| 2-Ethylpyrazine | 0.0376 | 0.0367 |
| 2,3-Dimethylpyrazine | 0.0121 | 0.0117 |
| 2-Methyl-2-cyclopentenone | 0.0016 | 0.0010 |
| 2-Ethyl-6-methylpyrazine | 0.0130 | 0.0126 |
| 2-Ethyl-5-methylpyrazine | 0.0084 | 0.0083 |
| 2,3,5-Trimethylpyrazine | 0.0097 | 0.0097 |
| 2-Ethyl-3-methylpyrazine | 0.0076 | 0.0078 |
| Propylpyrazine | 0.0290 | 0.0279 |
| Acetoin | 0.0269 | 0.0214 |
| Hexanal | 0.0009 | 0.0004 |
| 4-Ethylguaiacol | 0.0001 | 0.0000 |
| Pyrrole | 0.0087 | 0.0075 |
| Acetic acid | 0.4053 | 0.3336 |
| Furfural | 0.4159 | 0.3462 |
| Acetoxyacetone | 0.1181 | 0.0811 |
| 2-Fufurylmethyl sulfide | 0.0009 | 0.0005 |
| 2-Acetylfuran | 0.0358 | 0.0278 |
| 2-Ethyl-3,5-dimethylpyrazine | 0.0006 | 0.0005 |
| 2,3-Dimethyl-2-cyclopentenone | 0.0005 | 0.0003 |
| Acetoxy-2-butanone | 0.0169 | 0.0114 |
| 2-Furfurylacetate | 0.0255 | 0.0161 |
| Propionic acid | 0.0109 | 0.0080 |
| 3-Methylpyrrole | 0.0002 | 0.0002 |
| 5-Methylfurfural | 0.0831 | 0.0587 |
| 2-Acetylpyridine | 0.0006 | 0.0004 |
| 1-Methyl-2-formylpyrrole | 0.0033 | 0.0023 |
| g-Butyrolactone | 0.0095 | 0.0078 |
| Furfuryl alcohol | 0.2074 | 0.1813 |
| Isovaleric acid | 0.0293 | 0.0265 |
| 2-Furfuryl-5-methylfuran | 0.0001 | 0.0001 |
| 2,5-Dihydrofuranone | 0.0057 | 0.0056 |
| 1-Furfurylpyrrole | 0.0012 | 0.0008 |
| 2-Methoxy-4-vinylguaiacol | 0.0010 | 0.0008 |
| Phenylethyl alcohol | 0.0002 | 0.0002 |
| 2-Thiophenemethanol | 0.0003 | 0.0003 |
| 2-Acetylpyrrole | 0.0018 | 0.0018 |
| Difurfuryl ether | 0.0001 | 0.0001 |
| 2-Formylpyrrole | 0.0021 | 0.0018 |
| Pyridine | 0.0711 | 0.0480 |
| Guaiacol | 0.0005 | 0.0003 |
