# Supplementary material for: Impact of agro-forestry systems on the aroma generation of coffee beans
Source: Front Nutr. 2022 Aug 4;9:968783. doi: 10.3389/fnut.2022.968783 (PMC9386424; doi:10.3389/fnut.2022.968783)
Supplement: Supplementary file 8 [file Table_8.docx]

**Table 8 The quantitative data for volatile aroma compounds in the contrast group legume timber shade trees vs mom-legume timber shade trees**

|  | Legume  (mg/kg) | Non-legume  (mg/kg) |
| --- | --- | --- |
| 2-Methylfuran | 0.0200 | 0.0137 |
| p-Cresol | 0.0079 | 0.0054 |
| Diacetyl | 0.0534 | 0.0432 |
| 2,3-Pentanedione | 0.0938 | 0.0777 |
| Dimethyl Disulphide | 0.0029 | 0.0019 |
| 2-Vinylfuran | 0.0057 | 0.0039 |
| Vinylpyrazine | 0.0022 | 0.0019 |
| 2,3-Hexanedione | 0.0052 | 0.0032 |
| 1-Methylpyrrole | 0.0098 | 0.0070 |
| 2,5-Dimethylfuran | 0.0026 | 0.0017 |
| 2-Ethyl-3,6-dimethylpyrazine | 0.0027 | 0.0024 |
| 2,4,5-Trimethyloxazole | 0.0009 | 0.0007 |
| 2-Pentylfuran | 0.0003 | 0.0002 |
| 2-Methoxymethylfuran | 0.0016 | 0.0010 |
| 2-Methylpyrazine | 0.3174 | 0.2918 |
| Dihydro-2-methyl-3-furanone | 0.0525 | 0.0415 |
| 4-Methylthiazole | 0.0025 | 0.0021 |
| 2,6-Diethylpyrazine | 0.0004 | 0.0004 |
| 2,5-Dimethylpyrazine | 0.0333 | 0.0325 |
| 2,6-Dimethylpyrazine | 0.0694 | 0.0663 |
| 2-Ethylpyrazine | 0.0381 | 0.0347 |
| 2,3-Dimethylpyrazine | 0.0127 | 0.0127 |
| 2-Methyl-2-cyclopentenone | 0.0015 | 0.0010 |
| 2-Ethyl-6-methylpyrazine | 0.0133 | 0.0116 |
| 2-Ethyl-5-methylpyrazine | 0.0086 | 0.0079 |
| 2,3,5-Trimethylpyrazine | 0.0098 | 0.0087 |
| 2-Ethyl-3-methylpyrazine | 0.0078 | 0.0068 |
| Propylpyrazine | 0.0281 | 0.0273 |
| Acetoin | 0.0260 | 0.0214 |
| Hexanal | 0.0007 | 0.0004 |
| 4-Ethylguaiacol | 0.0001 | 0.0000 |
| Pyrrole | 0.0089 | 0.0075 |
| Acetic acid | 0.3843 | 0.3336 |
| Furfural | 0.3949 | 0.3462 |
| Acetoxyacetone | 0.1153 | 0.0811 |
| 2-Fufurylmethyl sulfide | 0.0009 | 0.0005 |
| 2-Acetylfuran | 0.0336 | 0.0278 |
| 2-Ethyl-3,5-dimethylpyrazine | 0.0006 | 0.0005 |
| 2,3-Dimethyl-2-cyclopentenone | 0.0005 | 0.0003 |
| Acetoxy-2-butanone | 0.0167 | 0.0114 |
| 2-Furfurylacetate | 0.0243 | 0.0161 |
| Propionic acid | 0.0103 | 0.0080 |
| 3-Methylpyrrole | 0.0002 | 0.0002 |
| 5-Methylfurfural | 0.0797 | 0.0587 |
| 2-Acetylpyridine | 0.0005 | 0.0004 |
| 1-Methyl-2-formylpyrrole | 0.0033 | 0.0023 |
| g-Butyrolactone | 0.0093 | 0.0078 |
| Furfuryl alcohol | 0.2043 | 0.1813 |
| Isovaleric acid | 0.0265 | 0.0257 |
| 2-Furfuryl-5-methylfuran | 0.0001 | 0.0001 |
| 2,5-Dihydrofuranone | 0.0056 | 0.0056 |
| 1-Furfurylpyrrole | 0.0011 | 0.0008 |
| 2-Methoxy-4-vinylguaiacol | 0.0010 | 0.0008 |
| Phenylethyl alcohol | 0.0002 | 0.0002 |
| 2-Thiophenemethanol | 0.0003 | 0.0003 |
| 2-Acetylpyrrole | 0.0018 | 0.0016 |
| Difurfuryl ether | 0.0001 | 0.0001 |
| 2-Formylpyrrole | 0.0022 | 0.0018 |
| Pyridine | 0.0719 | 0.0480 |
| Guaiacol | 0.0005 | 0.0003 |
